# Risk of malignant melanoma and colorectal cancer in Birt-Hogg-Dubé syndrome – a matched cohort study

**DOI:** 10.1038/s41416-025-03258-0

**Published:** 2025-11-05

**Authors:** Anna Skarin Nordenvall, Fredrik Persson, Anna Martling, Magnus Nordenskjöld, Maria Johansson Soller, Kristina Lagerstedt-Robinson, Giorgio Tettamanti, Fulya Taylan, Ann Nordgren

**Affiliations:** 1 Department of Molecular Medicine and Surgery, Karolinska Institutet, Stockholm, Sweden; 2 Department of Radiology, Karolinska University Hospital, Stockholm, Sweden; 3 Department of Clinical Genetics and Genomics, Sahlgrenska University Hospital, Gothenburg, Sweden; 4 Institute of Biomedicine, Department of Laboratory Medicine, University of Gothenburg, Gothenburg, Sweden; 5 Centre of Digestive Diseases, Karolinska University Hospital, Stockholm, Sweden; 6 Department of Clinical Genetics and Genomics, Karolinska University Hospital, Stockholm, Sweden; 7 Unit of Epidemiology, Institute of Environmental Medicine, Karolinska Institutet, Stockholm, Sweden

**Keywords:** Cancer epidemiology, Cancer genetics, Skin cancer

## Abstract

**Background:**

Birt-Hogg-Dubé syndrome (BHDS) is an autosomal dominant cancer predisposition syndrome, caused by pathogenic variants in *FLCN*, characterized by benign fibrofolliculomas, pulmonary cysts, pneumothorax, and increased risk for kidney tumors. To date, the risk of other solid tumors in individuals with BHDS remains unclear.

**Methods:**

We performed a register-based matched cohort study of 353 Swedish born individuals with verified pathogenic variants in *FLCN* to study the association between BHDS and other cancer types than kidney tumors. Each patient was matched to 50 comparisons, matched by birth year, birth county and sex. Associations were estimated using Cox Proportional Hazard models, expressed as Hazard Ratios with 95% Confidence Intervals.

**Results:**

Patients with BHDS exhibited a significantly elevated risk of adult cancer (adjusted HR 2.25, 95% CI 1.76-2.87), primarily driven by kidney tumors. Colorectal cancer and malignant melanoma were more prevalent in the BHDS cohort, reflected by a fivefold increase in risk of colorectal cancer (adjusted HR 5.44, 95% CI 2.74-10.81) and an almost threefold risk of malignant melanoma (adjusted HR 2.67, 95% CI 1.17-6.07).

**Conclusion:**

This study confirms the high risk of kidney tumors in BHDS and identifies increased risks for colorectal cancer and malignant melanoma, though the absolute risks remain low. Further research is needed to validate the colorectal cancer association and assess its implications for screening and management.

## Introduction

Birt-Hogg-Dubé syndrome (BHDS) (OMIM#135150) is a rare autosomal dominant tumor-predisposition syndrome caused by pathogenic variants in the *FLCN* gene encoding the folliculin protein which has been implicated in the regulation of cellular survival, proliferation, and growth by interacting with the mTOR signaling pathway [1, 2]. BHDS is characterized by benign fibrofolliculomas, lung cysts, spontaneous pneumothorax, and kidney tumors [3–5].

Approximately 300 pathogenic *FLCN* variants have been reported in more than 500 families with BHDS, and almost half of the individuals reported harbor c.1285del/c.1285dup variants. Based on reported individuals with personal and/or family history of BHDS related symptoms, an estimated prevalence of 1:200,000 has been proposed, with lower prevalence in some countries [5–10]. A recent study based on exome sequencing of 135,990 participants in a large healthcare system reported a 60 times higher prevalence where 1/3234 (0.03%) of the individuals had a pathogenic/likely pathogenic (P/LP) truncating variant in *FLCN* [11, 12].

The kidney tumors in BHDS are typically multifocal and predominantly fall into clear cell- and oncocytoma/hybrid types [5, 13]. Inactivation of the wildtype *FLCN* allele by a somatic second-hit in the kidneys of patients with BHDS cause kidney tumors and the gene is therefore known as a classical tumor suppressor [2, 14–16].

In addition to the risk of kidney tumors, other malignancies such as colorectal cancer (CRC), thyroid cancer, parotid oncocytoma and malignant melanoma have been described in BHDS [17–22]. Several reports have suggested that an increased risk of colorectal polyps and carcinomas might be associated with BHDS [15, 19, 21–24], but larger studies have not been able to confirm the association [25, 26]. On the tumor level, the role of *FLCN* in CRC in BHDS is largely unknown [26], few analyses of a somatic second-hit have been performed on tumor tissue from CRC in patients with BHDS, reporting conflicting results[10, 19]. Furthermore, conflicting risk estimates for melanoma in BHDS have been reported [15, 20, 22, 27, 28].

To date, no conclusive evidence for a genotype-phenotype correlation in *FLCN* variants have been established. The mutation hotspot c.1285 has been inconsistently linked to an increased risk of CRC [19, 26]. Conclusive evidence regarding the risk of CRC and malignant melanoma in BHDS patients is therefore of importance to identify true risk estimates and evaluate the need for clinical surveillance in this patient group.

In this register-based cohort study we investigate the risk of different cancer types other than kidney tumors in 353 individuals born in Sweden with genetically confirmed BHDS.

## Material and methods

### National registers

In Sweden, a unique personal identity number (PIN) assigned to permanent residents, enables linkage between extensive nationwide population- and hospital-based registers [29]. The Swedish National Board of Health and Welfare hold the National Patient Register (NPR) and the National Cancer Register (NCR). The NPR contain information on diagnoses given at hospitals and was established in 1964, reaching nationwide coverage by 1987 [30]. The NCR, established nationwide in 1958, contains information on all cases of cancers diagnosed in Sweden [31]. In both the NPR and NCR diagnoses are recorded in accordance with the International Classification of Diseases (ICD) or the International Classification of Diseases for Oncology (ICD-O). The clinical genetics department at Karolinska University Hospital holds the regional Laboratory Information Management Systems (LIMS) that includes genetic diagnoses and disease-causing variants on patients referred to Karolinska for genetic testing.

Demographic data of the Swedish population is collected and managed by Statistics Sweden [32].

### Study design, exposure, and outcome

The association between BHDS and cancer was studied by a matched cohort design. Exposure status was defined using the Karolinska LIMS, where all individuals with a *FLCN* mutation born in Sweden were selected. All patients were referred for targeted germline *FLCN* genetic testing due to clinical suspicion of BHDS or as relatives of individuals with genetically confirmed BHDS. None were identified through somatic analyses or referred for germline testing due to malignancies other than kidney. *FLCN* was not part of the gene panel for inherited cancers at Karolinska University Hospital during the study period.

For each patient, 50 comparisons, matched by birth year, birth county and sex, were acquired from Statistics Sweden. Diagnosis and date of pneumothorax and cancer were collected from the NPR and NCR (ICD-codes for pneumothorax are available in Supplementary Table 1). Cancer types were classified according to anatomical location and regarded as pediatric if diagnosed before the age of 20. CRC and malignant melanoma were sub-grouped as cancer in situ (CIS) or non-CIS according to morphology coding. Site specific cancer was analyzed both as first cancer event and specific cancer event ever. Demographic data was collected from Statistics Sweden.

### Statistical analysis

Associations were estimated using Cox proportional Hazards models with attained age as the underlying time scale, presented as Hazard Ratios (HR) with 95% Confidence Intervals (CIs). Individuals were followed from birth until outcome event, migration, death, or end of study period (2017-12-31). When analyzing adult cancer, individuals were considered at risk from 20 years of age. Schoenfeld’s residuals were used to test the proportionality hazards assumption. Analyses were performed as crude and adjusted for birth year, level of education, and sex.

To assess cancer risk in asymptomatic *FLCN* carriers, we conducted identical analyses on a “healthy *FLCN* carrier” cohort, which included individuals with a genetically confirmed *FLCN* mutation without a recorded diagnosis of kidney tumor or pneumothorax.

Analyses including less than five were censored for confidentiality. Statistical analyses were performed using Stata 16.1, the code supporting the findings of this study is available from the corresponding author upon reasonable request. Ethical approval was granted from the Stockholm Regional Ethics Committee, Sweden, all methods were performed in accordance with the relevant guidelines and regulations.

## Results

In total, 353 Swedish born individuals with pathogenic variants in the *FLCN*, analyzed at Karolinska University Hospital between 2002-2019, were identified through the Karolinska University Hospital LIMS, with equal numbers of affected men and women (Table 1), and a mean age of 48.25 ( ± 17.34) at end follow up. The mutation- and phenotype-spectrum has previously been reported in 186 of these patients [33].

**Table 1:** Characteristics of the study population.

	***Birt-Hogg-Dubé***	***Non Birt-Hogg-Dubé***
**N (%)**	353 (100.0)	17650 (100.0)
**Birth year**		
1930-1939	13 (3.7)	650 (3.7)
1940-1949	46 (13.0)	2300 (13.0)
1950-1959	56 (15.9)	2800 (15.9)
1960-1969	78 (22.1)	3900 (22.1)
1970-1979	57 (16.1)	2850 (16.1)
1980-1989	51 (14.4)	2550 (14.4)
1990-1999	43 (12.2)	2150 (12.2)
2000-2009	7 (2.0)	350 (2.0)
2010-2017	2 (0.6)	100 (0.6)
**Sex**		
Male	177 (50.1)	8850 (50.1)
Female	176 (49.9)	8800 (49.9)
**Educational level**		
Primary	36 (10.2)	2475 (14.0)
Secondary	148 (41.9)	8084 (45.8)
Post-secondary	163 (46.2)	6744 (38.2)
Missing	6 (1.7)	347 (2.0)
**Cancer**		
Cancer at any age, including CIS	71 (20.1)	1782 (10.1)
*mean age at first cancer (±SD)*	*47.39 (* ± *16.63)*	*49.30 (* ± *16.69)*
Cancer at any age, excluding CIS	52 (14.7)	1078 (6.1)
*mean age at first cancer (±SD)*	*51.17 (* ± *14.78)*	*55.80 (* ± *14.56)*
<20 y.a, excluding CIS	<5	22 (0.1)
*mean age at first cancer (±SD)*	*15.33 (* ± *5.51)*	*11.59 (* ± *6.28)*
Cancer >19 y.a, excluding CIS	49 (13.9)	1056 (6.0)
*mean age at first cancer (±SD)*	*53.37 (* ± *12.07)*	*56.72 (* ± *13.19)*
**Site-specific cancer (only first cancer event included, CIS excluded)**		
Colorectal	9 (2.5)	83 (0.5)
Lung	<5	33 (0.2)
Malignant Melanoma	<5	92 (0.5)
Other malignant skin tumors	<5	33 (0.2)
Kidney	21 (5.9)	27 (0.2)
Prostate	<5	220 (1.2)
Breast	<5	180 (1.0)
CNS	<5	41 (0.2)
Hematological	<5	84 (0.5)
**Emigrated**	17 (4.8)	219 (1.2)
*mean age at emigration (±SD)*	*30.83 (* ± *12.00)*	*32.90 (* ± *16.51)*
**Dead**	7 (2.0)	410 (2.3
*mean age at death (±SD)*	*66.58 (* ± *10.75)*	*68.14 (* ± *11.27)*
**Dec 31. 2017**	329 (93.2)	17021 (96.4)
*mean age at end of follow up (±SD)*	*48.25 (* ± *17.34)*	*48.66 (* ± *17.12)*

**Fig. 1:**
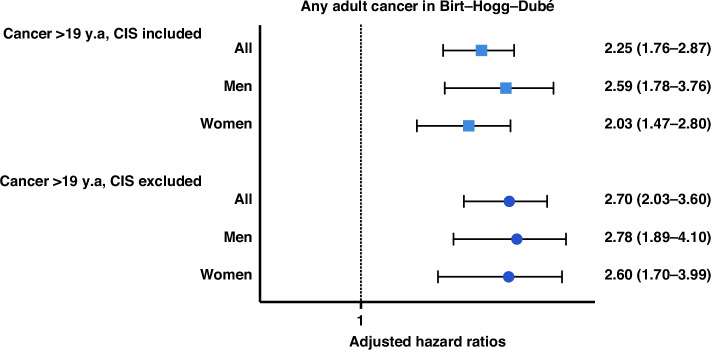
Hazard ratios for adult cancer in BHDS, adjusted for birth year and educational level, stratified by sex.

Cancer was diagnosed in 20% of BHDS-patients. The mean age at first cancer diagnosis was 47.39 ( ± 16.63) in BHDS-patients and 49.30 ( ± 16.69) in comparisons. Four BHDS patients were diagnosed with cancer before age 20, including two cases of papillary renal cell carcinoma. As indicated by descriptive data, there was an increased risk of adult cancer in patients with BHDS (aHR 2.25, 95% CI 1.76-2.87), with a similar increase in men and women (Fig. 1).

**Fig. 2:**
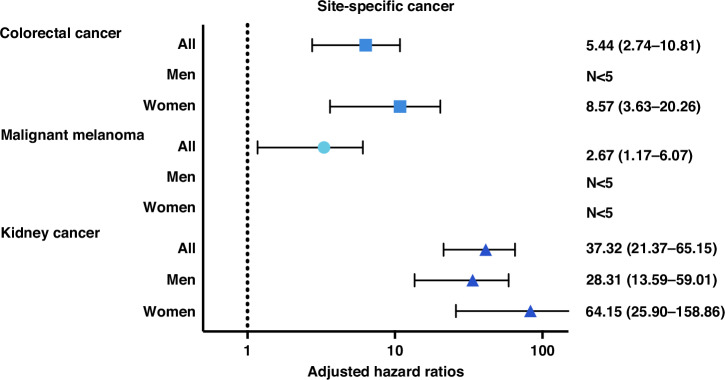
Hazard ratios for adult site-specific cancer in BHDS, CIS excluded. Adjusted for birth year and level of education, stratified by sex. Analyses on N < 5 not performed.

In analyses on site-specific cancer, 23 (6.5%) of the individuals with BHDS had a registered diagnosis of kidney tumor, compared to 0.2% in the matched comparison group, resulting in a heavily increased risk of kidney tumors (aHR 37.32, 95% CI 21.37-65.15) that was more pronounced in women than men (Fig. 2). Fifteen of the 23 individuals with kidney tumors were referred for genetic testing after their kidney tumor diagnosis. Among the kidney cancer events identified in the BHDS cohort, 10 (43.5%) were chromophobe renal cell carcinoma, 8 (34.8%) were clear cell renal cell carcinoma, and the remaining cases were papillary or unspecified malignant kidney cancers. Other cancer types observed in the BHDS cohort comprised melanoma, colorectal, connective tissue, hematological, adrenal, thyroid, peripheral nerve, non-melanoma skin, testicular, cervical, and breast cancers, with all types except melanoma and CRC being rare ( < 5 events) or occurring at frequencies comparable to the control cohort. Ten individuals with BHDS (3%) had a registered diagnosis of CRC as compared to 0.5% in the matched comparison group. There was an overall increased risk of CRC in the full cohort (aHR 5.44, 95% CI 2.74-10.81), particularly in women (aHR 8.57, 95% CI 3.63-20.26), risk in men could not be reliably assessed due to the low number of events (Fig. 2). The association remained significant after inclusion of colorectal CIS (aHR 4.53, 95% CI 2.37-8.64) (Supplementary Table 2). The mean age at diagnosis of CRC ( ± SD) was similar in BHDS and comparisons (59.4 ( ± 10.33) vs. 61.06 ( ± 10.86) years). Of the 10 patients with BHDS and CRC eight were diagnosed with CRC several years before BHDS.

Eight (2%) BHDS patients had melanoma, whereof 6 cases were non-CIS. Similar to CRC, most melanoma cases were diagnosed before BHDS was identified. When only taking first cancer event into account the risk of melanoma appeared equal in BHDS-patients and comparisons, however, when taking all individual cancer events into account results showed an increased risk of malignant melanoma in BHDS (aHR 2.67, 95% CI 1.17-6.07) (Supplementary Table 2).

In analyses of the “healthy *FLCN* carrier” cohort the overall cancer risk was diminished (aHR 1.75, 95% CI 1.06-2.88), a significant overall association with cancer remained in men, but not women (Supplementary Table 3). The risk of CRC was higher in the “healthy *FLCN* carrier” cohort, (aHR 8.07, 95% CI 3.39-19.22) than in the full cohort. All BHDS patients with melanoma had a registered diagnosis of kidney tumor or pneumothorax. (Supplementary Table 3).

Table 2 shows the distribution of cancer types and different pathogenic variants (PVs) according to the *FLCN* reference sequence NM_144997.7. One patient was excluded due to missing detailed mutation data. The Swedish founder splice donor variant, c.779+1 G > T, was present in 44% (156/352) of the cohort, whereof 19% (29/156) developed cancer. Among those with cancer, 40% (14/35) developed kidney tumors, 14% (5/35) developed CRC, and 9% (3/35) developed malignant melanoma.

**Table 2:** Distribution of cancer and PVs in the FLCN gene according to reference sequence NM_144997.7 in the BHDS population, grouped according to molecular function.

	Total no. individuals	Any cancer	No cancer	Total no. different cancer diagnoses	Kidney cancer	Colorectal cancer	Melanoma	Other cancer
	N	N (%)	N (%)	N	N (%)	N (%)	N (%)	N (%)
**PVs according to reference sequence NM_144997.7**								
**Deletion**	**15**	**3 (20)**	**12 (80)**	**3**	**0 (0)**	**1 (33,3)**	**1 (33,3)**	**1 (33,3)**
Deletion Exon 1	15	3 (20)	12 (80)	3	0 (0)	1 (33,3)	1 (33,3)	1 (33,3)
**Frameshift**	**142**	**30 (21)**	**112 (79)**	**32**	**6 (19)**	**4 (13)**	**3 (9)**	**19 (59)**
c.1285del (p.(His429Thrfs*39))	12	2 (17)	10 (83)	2	0 (0)	1 (50)	0 (0)	1 (50)
c.1285dup (p.(His429Profs*27))	62	19 (31)	43 (69)	19	5 (26)	2 (10,5)	2 (10,5)	10 (53)
c.1291dup (p.(Leu431Profs*25))	5	0 (0)	5 (100)	0	0 (0)	0 (0)	0 (0)	0 (0)
c.235_238del (p.(Ser79Thrfs*50))	50	9 (18)	41 (82)	11	1 (9)	1 (9)	1 (9)	8 (73)
c.241_242del (p.(Met81Valfs*18)))	11	0 (0)	11 (100)	0	0 (0)	0 (0)	0 (0)	0 (0)
Other	2	0 (0)	2 (100)	0	0 (0)	0 (0)	0 (0)	0 (0)
**Intron variant**	**7**	**1 (14)**	**6 (86)**	**1**	**0 (0)**	**0 (0)**	**0 (0)**	**1 (100)**
c.1177-5_1177-3del	7	1 (14)	6 (86)	1	0 (0)	0 (0)	0 (0)	1 (100)
**Splice acceptor**	**14**	**7 (50)**	**7 (50)**	**8**	**3 (37)**	**0 (0)**	**1 (13)**	**4 (50)**
c.1063-1 G > T	3	1 (33)	2 (67)	1	0 (0)	0 (0)	0 (0)	1 (100)
c.1301_1302del	9	6 (67)	3 (33)	7	3 (43)	0 (0)	1 (14)	3 (43)
Other	2	0 (0)	2 (100)	0	0 (0)	0 (0)	0 (0)	0 (0)
**Splice donor**	**156**	**29 (19)**	**127 (81)**	**35**	**14 (40)**	**5 (14)**	**3 (9)**	**13 (37)**
c.779+1 G > T	154	29 (19)	125 (81)	35	14 (40)	5 (14)	3 (9)	13 (37)
Other	2	0 (0)	2 (100)	0	0 (0)	0 (0)	0 (0)	0 (0)
**Missense**	**10**	**1 (10)**	**9 (90)**	**1**	0 (0)	0 (0)	0 (0)	**1 (100)**
c.764 A > C (p.His255Pro)	10	1 (10)	9 (90)	1	0 (0)	0 (0)	0 (0)	1 (100)
**Nonsense**	**8**	**0 (0)**	**8 (100)**	**0**	**0 (0)**	**0 (0)**	**0 (0)**	**0 (0)**
c.1429 C > T (p.Arg477Ter)	2	0 (0)	2 (100)	0	0 (0)	0 (0)	0 (0)	0 (0)
c.943 G > T (p.(Glu315Ter))	6	0 (0)	6 (100)	0	0 (0)	0 (0)	0 (0)	0 (0)

One patient excluded due to missing detailed mutation data. “Any cancer” indicates if an individual ever had cancer; “Total different cancer diagnoses” counts all distinct cancer events, including multiple per person.

Patients with frameshift variants formed the second largest group at 40% (142/352) of the cohort, with 52% (74/142) having either the c.1285del or c.1285dup variant. In this group, 21% (30/142) developed cancer, wherof 19% (6/32) developed kidney tumors, 13% (4/32) developed CRC, and 9% (3/32) developed malignant melanoma. Fifty individuals (35%, 50/142) carried the frameshift deletion c.235_238del, whereof 18% (9/50) developed cancer, with only one patient each developing CRC, malignant melanoma and kidney tumor.

Splice acceptor variants were present in 4% (14/352) of the cohort, with 64% (9/14) having the c.1301_1302del variant, whereof 50% (7/14) developed cancer. Among these tumors, three were kidney, and one was a malignant melanoma. Fifteen individuals (4%, 15/352) had deletion of exon one, and three of them developed cancer.

## Discussion

This matched cohort study of 353 Swedish born individuals with P/LP variants in *FLCN* confirms an increased risk of both CRC and malignant melanoma in patients with BHDS. The matched cohort study design, utilizing both Swedish National Register data and genetic data from the Karolinska University Hospital LIMS allows for long-term follow-up of mutational carriers and enables robust time-to-event analyses.

In our cohort, overall cancer penetrance was 20% at a mean age of 47.70 ( ± 16.56). Kidney tumor risk was significantly increased but exact risk estimates should be interpreted cautiously as the cohort was partly identified through on the presence of kidney tumors. The prevalence of kidney tumors in BHDS reported in the literature varies largely [34, 35], but was in a recent review of 204 BHDS families estimated to be 19% by the age of 70 [5]. Our observed lower prevalence may reflect a younger cohort.

Apart from the risk of kidney tumors, conclusive evidence for other tumor entities in BHDS is lacking. We found an overall 5-fold increased risk of CRC in BHDS. *FLCN* has previously been proposed to play a role in both familial and sporadic CRC susceptibility, regardless of BHDS [19, 36, 37]. Further, several reports have suggested that an increased risk of colorectal polyps and carcinomas might be associated with BHDS [10, 15, 19, 22–24, 38]. A recent study by Sattler and co-authors including 256 BHDS patients suggested an association with early onset of CRC due to a moderately but significantly increased rate of CRC [21]. These findings were contradicted in two Dutch and American studies based on 399 and 111 BHDS patients where no association between BHDS and colon neoplasms could be established [25, 26]. The differences between our results and the larger Dutch study may be partly due to the higher prevalence of CRC in their comparison group and possibly the differences in mean age (48.6 vs. 52.2). A recent systematic review including 221 individual *FLCN* carriers did not find an increased risk for colonic polyp*s* [5]. The higher HR for colorectal cancer observed in women compared with the full cohort may suggest an elevated underlying risk, but could also result from increased detection due to higher screening and surveillance participation. However, in this study direct sex-specific comparisons are limited by the small number of CRC events in men.

On the tumor level, the role of *FLCN* in CRCs in BHDS is largely unknown. Only a few somatic second-hit analyses have been performed on tumor tissue. Somatic poly(C)_8_ tract mutations in *FLCN* have previously been reported in CRC with demonstrated microsatellite instability (MSI+ tumors). However, no loss of the second *FLCN* allele was present in these tumors, and the *FLCN* mutations were suggested likely passenger variants caused by the MSI [26] or acting by a dominant negative effect [19], while a somatic second hit with loss of heterozygosity was detected in a patient with a germline *FLCN* c.1177-5_1177-3del mutation, implicating *FLCN* as an initiating factor in tumorigenesis in CRC [10].

We found a 2-3-fold increased risk of malignant melanoma, where 2% of our BHDS cohort had developed the disease at the end of follow-up. Previous studies on cutaneous and choroidal melanoma in BHDS patients have presented conflicting risk estimates [15, 20, 27]. In a study with a total of 83 BHDS patients an overall occurrence rate of 10% of melanoma and melanoma in situ was identified. This can be compared to the average lifetime risk estimates of cutaneous melanoma in the UK and the USA of 1.8%-2.4%, respectively [22]. Collectively, this data indicates an association between cutaneous malignant melanoma and BHDS. In a recent Finnish study of 270 patients with uveal melanoma none had pathogenic *FLCN* variants [28].

### Genotype phenotype correlations

No clear correlations between *FLCN* variants and specific phenotypes have been established, though some studies suggest different mutations may carry distinct risk profiles for cancer, lung cysts, and pneumothorax [34]. In our study, about 20% of carriers of splice donor, frameshift variants, and exon one deletions in *FLCN* developed cancer. The c.1285dup variant, previously linked to a lower risk of kidney tumors [13, 39], was found in various cancers in our cohort, including kidney tumors (26%), CRC (10.5%), and malignant melanoma (10.5%). Notably, 61% (14/23) of the kidney tumor cases had the Swedish founder variant c.779+1 G > T, also observed in 33% (13/39) of other cancer types, suggesting a stronger cancer predisposition compared to other variants. The c.1285dup variant has also been linked to a higher risk of CRC compared to other variants [19, 21, 39]. In our study, 10 individuals with pathogenic *FLCN* variants developed CRC, whereof 50% (5/10) were carrying the Swedish founder variant c.779+1 G > T, supporting its association with cancer predisposition. Additionally, eight individuals with pathogenic *FLCN* variants developed malignant melanoma. We cannot make any firm genotype-phenotypes correlations from our data. Further and larger studies are required to better define genotype-phenotype correlations in BHDS.

### Prevalence

Since not all Swedish patients were referred to Karolinska University Hospital for *FLCN* genetic analysis, our study does not cover all BHDS patients diagnosed in Sweden during the study period. Still, identifying 353 individuals with genetically confirmed *FLCN* variants suggests a prevalence of at least 35 cases per million. This aligns with the reported prevalence of 1 in 38,000 individuals in the Netherlands^12^, but is higher than the estimated prevalence of 1 in 200,000 individuals or 1.86 cases per million derived from epidemiological data on spontaneous pneumothorax [12, 40], or than the recent Korean study with 5.67 per 10 million [6]. A recent study based on a large health care population reported a much higher frequency of pathogenic *FLCN* variants (1 in 3,234 unrelated individuals), suggesting that the true cancer penetrance in BHDS may be lower than previously thought [11].

### Strengths

The high quality of the LIMS and NCR minimizes risk of misclassification for exposure and outcome. The design reduces selection- and recall bias, enables long-term follow-up of mutational carriers, and supports robust time-to-event analyses despite incomplete follow up. The large sample size and register-based cancer detection of cancer allow for reliable penetrance estimates of rare outcomes like malignant melanoma and CRC.

### Limitations

The cohort primarily included individuals and families referred for genetic testing due to BHDS manifestations, and may not reflect cancer risks in all *FLCN* mutation carriers. With a mean age under 50, lifetime risk of CRC and melanoma may be underestimated. Kidney tumor risk may be influenced by ascertainment bias, as many patients were referred after developing kidney tumors. Surveillance bias for CRC and melanoma is likely minimal since most patients were diagnosed with BHDS several years after their CRC or melanoma diagnoses. Detection bias from dermatologist refering patients with skin lesions is possible, but all melanoma cases had other BHDS manifestations like kidney tumors or pneumothorax. There is also potential detection bias for melanoma in BHDS patients seen by dermatologist due to fibrofolliculomas.

## Conclusion

This study indicates that pathogenic *FLCN* variants increase the risk of CRC and malignant melanoma, alongside the established risk of kidney tumors. However, the absolute risk of CRC and melanoma remains low. The 2024 guideline by Geilswijk et al. offers a robust framework for renal cancer screening in BHDS, emphasizing evidence-based surveillance and management strategies [41]. Building on this foundation, further studies in other BHDS cohorts are needed to confirm the elevated CRC risk before considering changes to screening guidelines. To understand the role of FLCN in the development of CRC and malignant melanoma, further paired tumor-germline second hit analyses could be valuable.

## Data avaliablity

Anonymized personal data were obtained from national Swedish Registry holders after ethical approval and secrecy assessment. According to Swedish laws and regulations, personal sensitive data can only be made available for researchers who fulfil legal requirements for access to personal sensitive data. Contact Professor Ann Nordgren (ann.nordgren@ki.se) for questions about data access.

## Supplementary information

Supplementary tables, non revised

## References

[1] Nickerson ML, Warren MB, Toro JR, Matrosova V, Glenn G, Turner ML, et al. Mutations in a novel gene lead to kidney tumors, lung wall defects, and benign tumors of the hair follicle in patients with the Birt-Hogg-Dubé syndrome. Cancer Cell. 2002;2:157–64. 12204536 10.1016/s1535-6108(02)00104-6

[2] Baba M, Hong SB, Sharma N, Warren MB, Nickerson ML, Iwamatsu A, et al. Folliculin encoded by the BHD gene interacts with a binding protein, FNIP1, and AMPK, and is involved in AMPK and mTOR signaling. Proc Natl Acad Sci USA. 2006;103:15552–7. 17028174 10.1073/pnas.0603781103 PMC1592464

[3] Birt AR, Hogg GR, Dubé WJ. Hereditary Multiple Fibrofolliculomas With Trichodiscomas and Acrochordons. Archives Dermatol. 1977;113:1674–7. 596896

[4] Toro JR, Glenn G, Duray P, Darling T, Weirich G, Zbar B, et al. Birt-Hogg-Dubé syndrome: a novel marker of kidney neoplasia. Arch Dermatol. 1999;135:1195–202. 10522666 10.1001/archderm.135.10.1195

[5] Bruinsma FJ, Dowty JG, Win AK, Goddard LC, Agrawal P, Attina’ D, et al. Update of penetrance estimates in Birt-Hogg-Dubé syndrome. J Med Genet. 2023;60:317–26. 36849229 10.1136/jmg-2022-109104

[6] Park HJ, Kim YJ, Kim MJ, Kim HC. Epidemiology and clinical features of Birt-Hogg-Dubé syndrome: A nationwide population-based study in South Korea. PLoS One. 2022;17:e0269358. 35666727 10.1371/journal.pone.0269358 PMC9170097

[7] Daccord C, Good JM, Morren MA, Bonny O, Hohl D, Lazor R. Birt-Hogg-Dubé syndrome. Eur Respir Rev. 2020;29:200042. 32943413 10.1183/16000617.0042-2020 PMC9489184

[8] Menko FH, van Steensel MAM, Giraud S, Friis-Hansen L, Richard S, Ungari S, et al. Birt-Hogg-Dubé syndrome: diagnosis and management. Lancet Oncol. 2009;10:1199–206. 19959076 10.1016/S1470-2045(09)70188-3

[9] Lim DHK, Rehal PK, Nahorski MS, Macdonald F, Claessens T, Van Geel M, et al. A new locus-specific database (LSDB) for mutations in the folliculin (FLCN) gene. Hum Mutat. 2010;31:E1043–1051. 19802896 10.1002/humu.21130

[10] Jirka GW, Lefler DS, Russo J, Bashir B. Colon adenocarcinoma and Birt-Hogg-Dubé syndrome in a young patient: case report and exploration of pathologic implications. Cancer Biol Ther. 2023;24:2184153. 36859772 10.1080/15384047.2023.2184153 PMC9988342

[11] Savatt JM, Shimelis H, Moreno-De-Luca A, Strande NT, Oetjens MT, Ledbetter DH, et al. Frequency of truncating FLCN variants and Birt-Hogg-Dubé-associated phenotypes in a health care system population. Genet Med. 2022;24:1857–66. 35639097 10.1016/j.gim.2022.05.006 PMC9703446

[12] van Riel L, Jansen PR, Boerrigter BG, van Moorselaar RJA, van Haelst MM, Wolthuis RMF, et al. Correspondence on “Frequency of truncating FLCN variants and Birt-Hogg-Dubé-associated phenotypes in a health care system population” by Savatt et al. Genet Med. 2023;25:158–60. 36383210 10.1016/j.gim.2022.08.033

[13] Sattler EC, Reithmair M, Steinlein OK. Kidney cancer characteristics and genotype-phenotype-correlations in Birt-Hogg-Dubé syndrome. PLoS One. 2018;13:e0209504. 30586397 10.1371/journal.pone.0209504 PMC6306193

[14] Hartman TR, Nicolas E, Klein-Szanto A, Al-Saleem T, Cash TP, Simon MC, et al. The role of the Birt-Hogg-Dubé protein in mTOR activation and renal tumorigenesis. Oncogene. 2009;28:1594–604. 19234517 10.1038/onc.2009.14 PMC2664853

[15] Khoo SK, Giraud S, Kahnoski K, Chen J, Motorna O, Nickolov R, et al. Clinical and genetic studies of Birt-Hogg-Dubé syndrome. J Med Genet. 2002;39:906–12. 12471204 10.1136/jmg.39.12.906 PMC1757219

[16] Vocke CD, Yang Y, Pavlovich CP, Schmidt LS, Nickerson ML, Torres-Cabala CA, et al. High frequency of somatic frameshift BHD gene mutations in Birt-Hogg-Dubé-associated renal tumors. J Natl Cancer Inst. 2005;97:931–5. 15956655 10.1093/jnci/dji154

[17] Chung JY, Ramos-Caro FA, Beers B, Ford MJ, Flowers F. Multiple lipomas, angiolipomas, and parathyroid adenomas in a patient with Birt-Hogg-Dube syndrome. Int J Dermatol. 1996;35:365–7. 8734663 10.1111/j.1365-4362.1996.tb03642.x

[18] Liu V, Kwan T, Page EH. Parotid oncocytoma in the Birt-Hogg-Dubé syndrome. J Am Acad Dermatol. 2000;43:1120–2. 11100034 10.1067/mjd.2000.109288

[19] Nahorski MS, Lim DHK, Martin L, Gille JJP, McKay K, Rehal PK, et al. Investigation of the Birt-Hogg-Dube tumour suppressor gene (FLCN) in familial and sporadic colorectal cancer. J Med Genet. 2010;47:385–90. 20522427 10.1136/jmg.2009.073304

[20] Cocciolone RA, Crotty KA, Andrews L, Haass NK, Moloney FJ. Multiple desmoplastic melanomas in Birt-Hogg-Dubé syndrome and a proposed signaling link between folliculin, the mTOR pathway, and melanoma susceptibility. Arch Dermatol. 2010;146:1316–8. 21079084 10.1001/archdermatol.2010.333

[21] Sattler EC, Syunyaeva Z, Reithmair M, Dempke W, Steinlein OK. Colorectal cancer risk in families with Birt-Hogg-Dubé syndrome increased. Eur J Cancer. 2021;151:168–74. 34000505 10.1016/j.ejca.2021.04.013

[22] Sattler EC, Ertl-Wagner B, Pellegrini C, Peris K, Reithmair M, Schädle N, et al. Cutaneous melanoma in Birt-Hogg-Dubé syndrome: part of the clinical spectrum?. Br J Dermatol. 2018;178:e132–3. 28869776 10.1111/bjd.15937

[23] Hornstein OP, Knickenberg M. Perifollicular fibromatosis cutis with polyps of the colon–a cutaneo-intestinal syndrome sui generis. Arch Dermatol Res. 1975;253:161–75. 1975 Sep 12. 1200700 10.1007/BF00582068

[24] Rongioletti F, Hazini R, Gianotti G, Rebora A. Fibrofolliculomas, tricodiscomas and acrochordons (Birt-Hogg-Dubé) associated with intestinal polyposis. Clin Exp Dermatol. 1989;14:72–4. 2805394 10.1111/j.1365-2230.1989.tb00890.x

[25] Zbar B, Alvord WG, Glenn G, Turner M, Pavlovich CP, Schmidt L, et al. Risk of renal and colonic neoplasms and spontaneous pneumothorax in the Birt-Hogg-Dubé syndrome. Cancer Epidemiol Biomark Prev. 2002;11:393–400. 11927500

[26] van de Beek I, Glykofridis IE, Wolthuis RMF, Gille HJJP, Johannesma PC, Meijers-Heijboer HEJ, et al. No evidence for increased prevalence of colorectal carcinoma in 399 Dutch patients with Birt-Hogg-Dubé syndrome. Br J Cancer. 2020;122:590–4. 31857718 10.1038/s41416-019-0693-1 PMC7028712

[27] Fontcuberta IC, Salomão DR, Quiram PA, Pulido JS. Choroidal melanoma and lid fibrofoliculomas in Birt-Hogg-Dubé syndrome. Ophthalmic Genet. 2011;32:143–6. 21401403 10.3109/13816810.2010.544367

[28] Repo PE, Jakkula E, Hiltunen J, Putkuri H, Staskiewicz-Tuikkanen A, Järvinen RS, et al. Pathogenic Germline Variants in Uveal Melanoma Driver and BAP1-Associated Genes in Finnish Patients with Uveal Melanoma. Pigment Cell Melanoma Res. 2024; 10.1111/pcmr.13198 PMC11681845 39344744

[29] Ludvigsson JF, Otterblad-Olausson P, Pettersson BU, Ekbom A. The Swedish personal identity number: possibilities and pitfalls in healthcare and medical research. Eur J Epidemiol. 2009;24:659–67. 19504049 10.1007/s10654-009-9350-y PMC2773709

[30] Ludvigsson JF, Andersson E, Ekbom A, Feychting M, Kim JL, Reuterwall C, et al. External review and validation of the Swedish national inpatient register. BMC Public Health. 2011;11:450. 21658213 10.1186/1471-2458-11-450 PMC3142234

[31] Barlow L, Westergren K, Holmberg L, Talbäck M. The completeness of the Swedish Cancer Register – a sample survey for year 1998. Acta Oncologica. 2009;48:27–33. 18767000 10.1080/02841860802247664

[32] Ludvigsson JF, Almqvist C, Bonamy AKE, Ljung R, Michaëlsson K, Neovius M, et al. Registers of the Swedish total population and their use in medical research. Eur J Epidemiol. 2016;31:125–36. 26769609 10.1007/s10654-016-0117-y

[33] Lagerstedt-Robinson K, Baranowska Körberg I, Tsiaprazis S, Björck E, Tham E, Poluha A, et al. A retrospective two centre study of Birt-Hogg-Dubé syndrome reveals a pathogenic founder mutation in FLCN in the Swedish population. PLoS One. 2022;17:e0264056. 35176117 10.1371/journal.pone.0264056 PMC8853502

[34] Toro JR, Wei MH, Glenn GM, Weinreich M, Toure O, Vocke C, et al. BHD mutations, clinical and molecular genetic investigations of Birt-Hogg-Dubé syndrome: a new series of 50 families and a review of published reports. J Med Genet. 2008;45:321–31. 18234728 10.1136/jmg.2007.054304 PMC2564862

[35] Kunogi M, Kurihara M, Ikegami TS, Kobayashi T, Shindo N, Kumasaka T, et al. Clinical and genetic spectrum of Birt-Hogg-Dube syndrome patients in whom pneumothorax and/or multiple lung cysts are the presenting feature. J Med Genet. 2010;47:281–7. 20413710 10.1136/jmg.2009.070565 PMC2981024

[36] Targeted sequencing of 36 known or putative colorectal cancer susceptibility genes - DeRycke - 2017 - Molecular Genetics & Genomic Medicine - Wiley Online Library [Internet]. [cited 2024 Sep 11]. Available from: https://onlinelibrary-wiley-com.proxy.kib.ki.se/doi/10.1002/mgg3.317 10.1002/mgg3.317 PMC5606870 28944238

[37] Dobbins SE, Broderick P, Chubb D, Kinnersley B, Sherborne AL, Houlston RS. Undefined familial colorectal cancer and the role of pleiotropism in cancer susceptibility genes. Familial Cancer. 2016;15:593–9. 27356891 10.1007/s10689-016-9914-4 PMC5010824

[38] Binet O, Robin J, Vicart M, Ventura G, Beltzer-Garelly E. Fibromes périfolliculaires, polypose colique familiale, pneumothorax spontanés familiaux. Ann Dermatol Venereol. 1986;113:928–30.

[39] Schmidt LS, Linehan WM. Molecular genetics and clinical features of Birt-Hogg-Dubé syndrome. Nat Rev Urol. 2015;12:558–69. 26334087 10.1038/nrurol.2015.206 PMC5119524

[40] Muller ME, Daccord C, Taffé P, Lazor R. Prevalence of Birt-Hogg-Dubé Syndrome Determined Through Epidemiological Data on Spontaneous Pneumothorax and Bayes Theorem. Front Med (Lausanne). 2021;8:631168. 33987191 10.3389/fmed.2021.631168 PMC8111214

[41] Geilswijk M, Genuardi M, Woodward ER, Nightingale K, Huber J, Madsen MG, et al. ERN GENTURIS clinical practice guidelines for the diagnosis, surveillance and management of people with Birt-Hogg-Dubé syndrome. Eur J Hum Genet. 2024;32:1542–50. 39085584 10.1038/s41431-024-01671-2 PMC11607457

