# Supplementary material for: Risk of malignant melanoma and colorectal cancer in Birt-Hogg-Dubé syndrome – a matched cohort study
Source: Br J Cancer. 2025 Nov 5;134(2):245–51. doi: 10.1038/s41416-025-03258-0 (PMC12820364; doi:10.1038/s41416-025-03258-0)
Supplement: Supplementary file 1 — Supplementary tables, non revised [file 41416_2025_3258_MOESM1_ESM.docx]

Supplementary Material

***Supplementary table 1.*** *List of ICD-codes for identifying spontaneous pneumothorax.*

**ICD-system ICD-code Codes for pneumothorax**

| ICD-7 | 520.99 |
| --- | --- |
| ICD-8 | 512.99 |
| ICD-9 | 512 |
| ICD-10 | J93 |

**Supplementary table 2.** Hazard ratios for adult and site-specific cancer in BHD, crude and adjusted for *birth year, parental level of education and sex, or **birth year and parental level of education. N less than five are censored.

***l***

|  | | ***Al*** |  |  | ***Men*** |  |  | ***Women*** |  |
| --- | --- | --- | --- | --- | --- | --- | --- | --- | --- |
|  | **N** | **Crude HR (95% CI)** | **Adjusted* HR (95% CI)** | **N** | **Crude HR (95% CI)** | **Adjusted** HR (95% CI)** | **N** | **Crude HR (95% CI)** | **Adjusted** HR (95% CI)** |
| **Any cancer** |  |  |  |  |  |  |  |  |  |
| Cancer >19 y.a, including CIS | 67 | 2.25 (1.76-2.87) | 2.25 (1.76-2.87) | 29 | 2.63 (1.82-3.82) | 2.59 (1.78-3.76) | 38 | 2.03 (1.47-2.81) | 2.03 (1.47-2.80) |
| Cancer >19 y.a, excluding CIS | 49 | 2.78 (2.09-3.70) | 2.70 (2.03-3.60) | 27 | 2.85 (1.94-4.20) | 2.78 (1.89-4.10) | 22 | 2.64 (1.72-4.04) | 2.60 (1.70-3.99) |
|  |  |  |  |  |  |  |  |  |  |
| **Site-specific cancer** |  |  |  |  |  |  |  |  |  |
| Colorectal cancer including CIS |  |  |  |  |  |  |  |  |  |
| *As first cancer* | 10 | 4.95 (2.59-9.46) | 5.13 (2.68-9.82) |  | N.A |  | 7 | 7.99 (3.62-17.62) | 7.86 (3.55-17.38) |
| *Ever* | 10 | 4.37 (2.30-8.32) | 4.53 (2.37-8.64) |  | N.A |  | 7 | 6.90 (3.15-15.11) | 6.92 (3.15-15.21) |
| Colorectal cancer excluding CIS |  |  |  |  |  |  |  |  |  |
| *As first cancer* | 9 | 6.04 (3.04-12.03) | 6.17 (3.09-12.31) |  | N.A |  | 6 | 9.54 (4.02-22.66) | 9.61 (4.03-22.89) |
| *Ever* | 9 | 5.34 (2.69-10.59) | 5.44 (2.74-10.81) |  | N.A |  | 6 | 8.39 (3.56-19.77) | 8.57 (3.63-20.26) |
|  |  |  |  |  |  |  |  |  |  |
| Melanoma including CIS |  |  |  |  |  |  |  |  |  |
| *As first cancer* | 5 | 1.87 (0.76-4.55) | 1.76 (0.72-4.28) |  | N.A |  |  | N.A |  |
| *Ever* | 8 | 2.50 (1.23-5.08) | 2.36 (1.16-4.80) |  | N.A |  | 5 | 2.67 (1.09-6.56) | 2.51 (1.02-6.16) |
| Melanoma excluding CIS |  |  |  |  |  |  |  |  |  |
| *As first cancer* | <5 |  | N.A |  | N.A |  |  | N.A |  |
| *Ever* | 6 | 2.91 (1.28-6.62) | 2.67 (1.17-6.07) |  | N.A |  |  | N.A |  |
|  |  |  |  |  |  |  |  |  |  |
| Kidney cancer as first cancer | 21 | 39.48 (21.85-71.33) | 41.41 (22.82-75.14) | 13 | 29.14 (13.96-60.82) | 31.74 (15.02-67.06) | 8 | 74.02 (25.68-213.35) | 75.83 (26.18-219.61) |
| Kidney cancer ever | 23 | 36.47 (20.96-63.47) | 37.32 (21.37-65.15) | 13 | 26.53 (12.86-54.72) | 28.31 (13.59-59.01) | 10 | 60.96 (24.76-150.07) | 64.15 (25.90-158.86) |

**Supplementary table 3.** Hazard ratios for adult and CRC cancer in BHDS without registered diagnosis of pneumothorax or kidney cancer. Results are presented as crude and adjusted for *birth year, level of education and sex. N less than five are censored.

|  |  | ***All*** |  |  | ***Men*** |  |  | ***Women*** | |
| --- | --- | --- | --- | --- | --- | --- | --- | --- | --- |
|  | **N** | **Crude HR (95% CI)** | **Adjusted* HR (95% CI)** | **N** | **Crude HR (95% CI)** | **Adjusted** HR (95% CI)** | **N** | **Crude HR (95% CI)** | **Adjusted** HR (95% CI)** |
| **Any cancer** |  |  |  |  |  |  |  |  |  |
| Cancer >19 y.a, including CIS | 28 | 1.77 (1.21-2.57) | 1.77 (1.21-2.57) | 11 | 2.34 (1.28-4.28) | 2.37 (1.30-4.34) | 17 | 1.56 (0.96-2.53) | 1.55 (0.96-2.52) |
| Cancer >19 y.a, excluding CIS | 16 | 1.76 (1.07-2.90) | 1.75 (1.06-2.88) | 9 | 2.18 (1.12-4.24) | 2.21 (1.13-4.31) | 7 | 1.45 (0.69-3.07) | 1.44 (0.68-3.05) |
|  |  |  |  |  |  |  |  |  |  |
| **Site-specific cancer** |  |  |  |  | |  | | | |
| Colorectal cancer including CIS |  |  |  |  | |  | | | |
| *As first cancer* | 6 | 5.99 (2.58-13.96) | 5.85 (2.50-13.68) | N.A | | N.A | | | |
| *Ever* | 6 | 5.28 (2.28-12.22) | 5.08 (2.19-11.82) | N.A | | N.A | | | |
| Colorectal cancer excluding CIS |  |  |  |  | |  | | | |
| *As first cancer* | 6 | 9.31 (3.90-22.18) | 9.29 (3.88-22.27) | N.A | | N.A | | | |
| *Ever* | 6 | 8.36 (3.53-19.79) | 8.07 (3.39-19.22) | N.A | | N.A | | | |
|  |  |  |  |  | |  | | | |
